# Identification of molecular glues of the SLP76/14-3-3 protein–protein interaction†

**DOI:** 10.1039/d1md00172h

**Published:** 2021-08-02

**Authors:** Lorenzo Soini, Martin Redhead, Marta Westwood, Seppe Leysen, Jeremy Davis, Christian Ottmann

**Affiliations:** Laboratory of Chemical Biology, Department of Biomedical Engineering and Institute for Complex Molecular Systems, Eindhoven University of Technology Eindhoven The Netherlands C.Ottmann@tue.nl; Department of Chemistry, UCB Celltech Slough UK; Exscientia Ltd, Schrodinger Building, Oxford Science Park Oxford OX44GE UK; Structural Biology, Discovery, Charles River, Chesterford Research Park UK; Department of Structural Biology and Biophysics, UCB Celltech Slough UK

## Abstract

The stabilisation of protein–protein interactions (PPIs) through molecular glues is a novel and promising approach in drug discovery. In stark contrast to research in protein–protein inhibition the field of stabilisation remains underdeveloped with comparatively few examples of small-molecule stabilisers of PPIs reported to date. At the same time identifying molecular glues has received recent sustained interest, especially in the fields of targeted protein degradation and 14-3-3 PPIs. The hub-protein 14-3-3 has a broad interactome with more than 500 known protein partners which presents a great opportunity for therapeutic intervention. In this study we have developed an HTRF assay suitable for HTS of the 14-3-3/SLP76 PPI and have completed a proof of concept screen against a chemically diverse library of 20 K molecules. The adaptor protein SLP76 has been reported to interact with 14-3-3 proteins downstream of the TCR playing an important role in mediating its own proteasomal degradation. We believe that stabilisation of this PPI could be exploited to potentiate degradation of SLP76 and therefore inhibit TCR signalling. This would represent an interesting alternative to other approaches in the field of targeted protein degradation. Here we disclose 16 novel stabilisers of the 14-3-3/SLP76 PPI across multiple different chemotypes. Based on the early results presented here we would recommend this approach to find molecular glues with broad applicability in the field of 14-3-3 PPIs.

## Abstract 2

The stabilisation of protein–protein interactions (PPIs) through molecular glues is a novel and promising approach in drug discovery.

## Graphical abstract

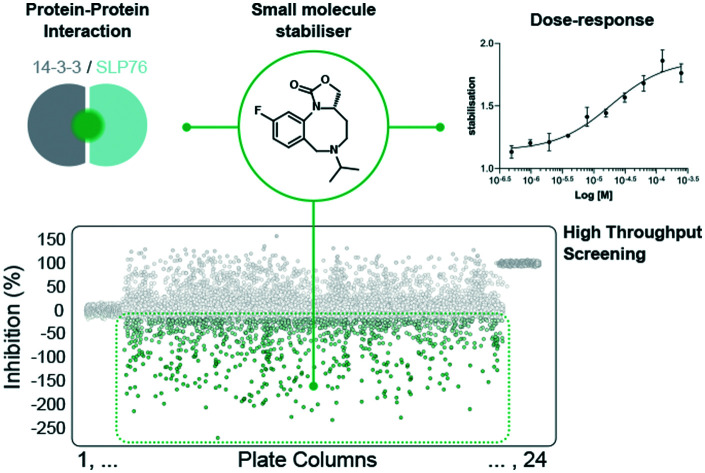

## Introduction

Inhibition of protein–protein interactions has become an established approach in drug discovery and has delivered successful examples to the market and in clinical trials.^1–3^ In contrast, the stabilisation of protein–protein interactions (PPI) remains a relatively novel field in drug discovery but has seen a considerable increase in interest over the last two decades. Although stabilising PPIs is considered a challenging approach, it has significant advantages. The binding epitopes generated at the interface of two different target surfaces confer an intrinsically higher potential for selectivity and theoretically even modest enhancements of the affinity of an already occurring PPI could lead to useful levels of activity.^4^

The natural immunosuppressant products cyclosporine and FK506 were among the first reported examples of molecular glues, although their mechanism of action was elucidated retrospectively.^5–7^ Most recently, small-molecule approaches have been pursued. PROTACs (proteolysis targeting chimeras) are classes of bifunctional small molecules that promote proximity between an ubiquitin ligase and a substrate protein, leading to the degradation of the latter.^8–11^ Smaller, monovalent molecules grouped under the IMiDs drug class, capable of promoting the ubiquitination of substrates targeting them for degradation, have also been described with thalidomide and lenalidomide the main examples.^12–15^ The ability to target proteins for degradation using small molecules has expanded the range of accessible new targets previously considered to be undruggable.^16,17^

So far, rationally designed small molecules aimed at the stabilisation of a PPI between an E3 ligase and its specific interaction partner has been the direct route to enhance protein degradation.^18–21^ However, mechanisms that indirectly promote protein degradation might be an alternate approach to find novel, therapeutically useful chemical matter.

Protein degradation can be modulated *via* ubiquitination,^22^ phosphorylation,^23,24^ acetylation,^25^ sumoylation,^26^ or, as some studies have suggested, *via* interaction with adaptor proteins such as 14-3-3.^27–31^ 14-3-3 proteins are a family of eukaryotic adaptor proteins that are known for recognizing specific pSer/pThr containing motifs on binding partners.^32,33^ In humans, they are present in seven isoforms (β, γ, σ, ζ, η, ε and τ). Composed of alpha helices and loops they can form either homo and hetero dimers.^34^ 14-3-3 proteins have a remarkable interactome with up to 500 binding partners^35^ and are involved in a plethora of biological processes which include apoptosis, cellular trafficking, cell-cycle and signal transduction.^36^ With a role as a central hub protein this makes the 14-3-3 family drug targets of huge, but as yet unrealised, potential.^37^ Among the most important related diseases to 14-3-3 and therapeutically relevant binding partners are: cancer (p53),^38^ Parkinson's disease (LRRK2),^39,40^ Alzheimer's disease (Tau)^41^ and cystic fibrosis (CTRF).^42,43^ The stabilisation of 14-3-3 PPIs has been an area of active research since the natural product fusicoccin-A (FC-A), a wilt-inducing phytotoxin produced by the fungus *Phomopsis amygdali*, was first identified as a 14-3-3/H^+^-ATPase PPI stabiliser.^44,45^ Cotylenin-A (CN-A), a remarkably similar compound to FC-A has also been reported to stabilise 14-3-3 PPIs.^46,47^ The natural products fusicoccin-A and cotylenin-A showed very good stabilisation properties and physiological activity on the 14-3-3/H^+^-ATPase system, theoretically opening the way for natural products derivatives that could improve binding properties and introduce selectivity for other systems.^45,47,48^ However, their chemical complexity and properties make them challenging to progress as potential drug candidates. Biosynthesis, total synthesis and semi-synthesis are under investigation, but are far from being completely actualised.^49–51^ Pyrroldone-1 was the first 14-3-3 stabiliser identified *via* high throughput screening (HTS), providing the possibility of more easily optimisable starting points amenable to rational design.^52,53^ Fragment-based approaches have also been pursued^54–56^ Despite these successful examples of 14-3-3 PPI stabilisers the field is still significantly lacking in novel chemotypes to stimulate development of the next generation of PPI stabilisers. In this study we have developed a homogenous time-resolved fluorescence (HTRF) assay suitable for HTS on the 14-3-3/SLP76 PPI and have completed a proof of concept screen against a chemically diverse library of 20 K molecules. Previous screening approaches have relied on short phosphopeptides to mimic the 14-3-3 protein partner which, although very useful, have important limitations.^52,54,56–58^ The employment of short synthetic peptides that mimic a much larger protein entity in fact greatly reduces the contact surface generated by the PPI. Subsequently, areas of the PPI that could serve as potential binding pockets are missing. Pivotal to our approach was to commit to using an SLP76 protein construct which we believe is both more relevant and opens the possibility of finding molecular glues beyond the small binding epitope of the peptide.

14-3-3 has been reported to interact with the protein SLP76 *via* one specific phosphorylation site located on SLP76, Ser376.^59,60^ SLP76 is an adaptor protein that orchestrates the signalling downstream of TCRs helping to modulate the immune response.^61,62^ The SLP76 phosphorylation on Ser376 is performed by the kinase HPK1 (hematopoietic progenitor kinase 1) and revealed a sophisticated negative feedback mechanism by which TCR signalling is modulated. The binding of 14-3-3 to SLP76 seems to mediate the proteasomal degradation of the latter which results in a negative regulation of TCR signalling. We believe that stabilisation of the 14-3-3/SLP76 PPI by means of a small molecule could therefore increase SLP76 degradation. An enhancement of the negative regulation of the TCR signalling could be beneficial in the context of autoimmune and inflammatory conditions mainly driven by over activated T-cells.

In order to find stabilisers of the 14-3-3/SLP76 PPI we generated a protein system composed of full-length 14-3-3γ and a 20 KDa phosphorylated SLP76 construct. This introduced a higher degree of complexity since 14-3-3 PPIs are more typically investigated using 12-mer synthetic peptides to mimic the 14-3-3 binding partner. We then developed and ran an HTRF-HTS (homogeneous time resolved fluorescence high throughput screening) on the UCB “Diversity Set” of 20 000 small molecules. The “Diversity Set” is a subset of the larger UCB HTS compound collection specifically selected for lead-like molecular properties and chemical diversity. We also used an unrelated PPI that shared the same FRET pair as the 14-3-3γ/SLP76 system as a counterscreen, which allowed us to identify specific potential stabilisers of this interaction. The hits found were further characterised performing HTRF dose–response and dose-ratio assays and the stabilisation effect orthogonally confirmed by SPR.

## Results and discussion

### The 14-3-3γ/SLP76 PPI and HTRF assay set up description

**Fig. 1:**
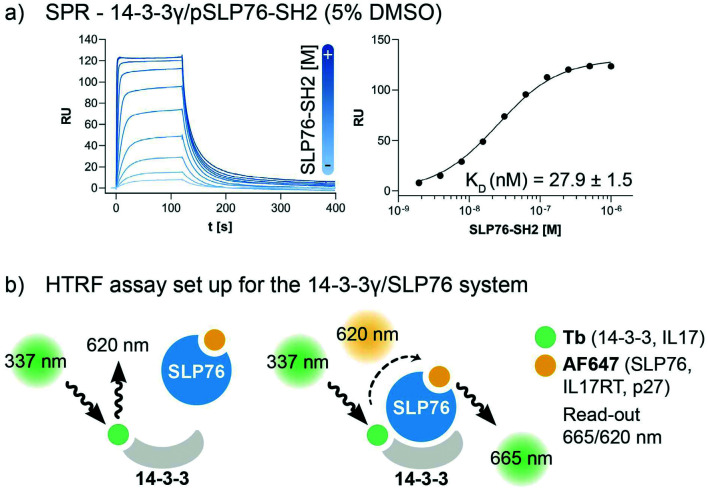
Assay set-up for the 14-3-3γ/SLP76 PPI. a) SPR binding assay of the SLP76-SH2 construct binding to 14-3-3γ in the presence of 5% DMSO. The *K*_D_ value has been extrapolated from the binding curve reported on the right of the sensorgrams. b) HTRF assay set-up. The two proteins of interest, SLP76 and 14-3-3γ have been labelled with Tb and AF647, respectively, to generate a suitable matched pair of donor molecule (Tb) and acceptor molecule (AF647). The donor molecule, Tb labelled 14-3-3, is excited at the proper wavelength of 337 nm. When the acceptor is not in the proximity (no binding) no signal is detected. When SLP76 bound to 14-3-3 brings the acceptor molecule AF647 close to the donor, energy transfer occurs between the fluorophores pair. A radiation with the wavelength of 665 nm is consequently emitted to generate a read-out (665/620 nm). All HTRF protein systems used in this work are reported in the legend.

The interaction between 14-3-3 proteins and SLP76 has been previously characterized exploiting a short synthetic phosphorylated peptide, SLP76pS376.^63^ Fluorescence polarization binding assay identified 14-3-3γ as the isoform with highest affinity against such system.^63^ Moreover, relative affinities of cellular SLP76 binding to 14-3-3 protein also report 14-3-3γ as the highest affinity isoform highlighting its biological importance.^27^ 14-3-3γ was therefore chosen as the isoform to perform the screening against. A detailed characterisation of the 14-3-3γ/SLP76 PPI with the exact SLP76 construct used here has already been described in another work.^63^ However, an additional SPR binding assay in the presence of 5% DMSO from which the *K*_D_ is calculated is reported (Fig. 1a).

**Fig. 2:**
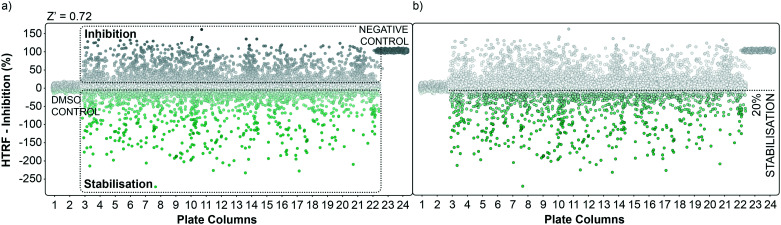
Graphical representation of the high throughput screening performance. a) Dots representation of the HTS in the plate format. Potential inhibitors are represented as grey dots, potential stabilisers are represented as green dots. DMSO control and negative control are grouped in plate columns 1, 2 and 23, 24 respectively. The calculated overall *Z*′ factor for the assay was 0.72. b) An initial cut-off of 20% stabilisation was used to select hits to follow-up.

The HTRF assay was set up by labelling 14-3-3γ and SLP76 with a matched pair of fluorophores. 14-3-3γ was labelled with Tb, generating the donor molecule, and SLP76 with AF647, generating the acceptor molecule. The read-out in HTRF originates by the proximity of the two proteins resulting in a FRET signal, which is directly proportional to the amount and strength of the interacting proteins (Fig. 2a).

### Primary HTRF HTS screening of 20 000 small molecules against the 14-3-3γ/SLP76 PPI

The performance of the initial single point screening of the 20 000 molecule UCB Diversity Deck against the 14-3-3γ/SLP76 PPI system is shown in (Fig. 2a). The peptide R18 is a well characterised inhibitor of 14-3-3 PPIs^64,65^ and was therefore chosen as the assay negative control and was also an important reagent used during assay validation. Unfortunately, there are no reported stabilisers of 14-3-3/SLP76 interactions and therefore no true positive controls. Of course, the fundamental aim of this work was to discover molecular glues of this interaction which can then be used as starting points and tool compounds to evaluate the therapeutic potential of stabilising the complex. The cut-off for selection of hits in the first round of screening was set at 20% stabilisation (or −20% inhibition) at a concentration of 100 μM with the reference control set at 100% inhibition and all the screening outcomes normalised accordingly (Fig. 2b).

**Fig. 3:**
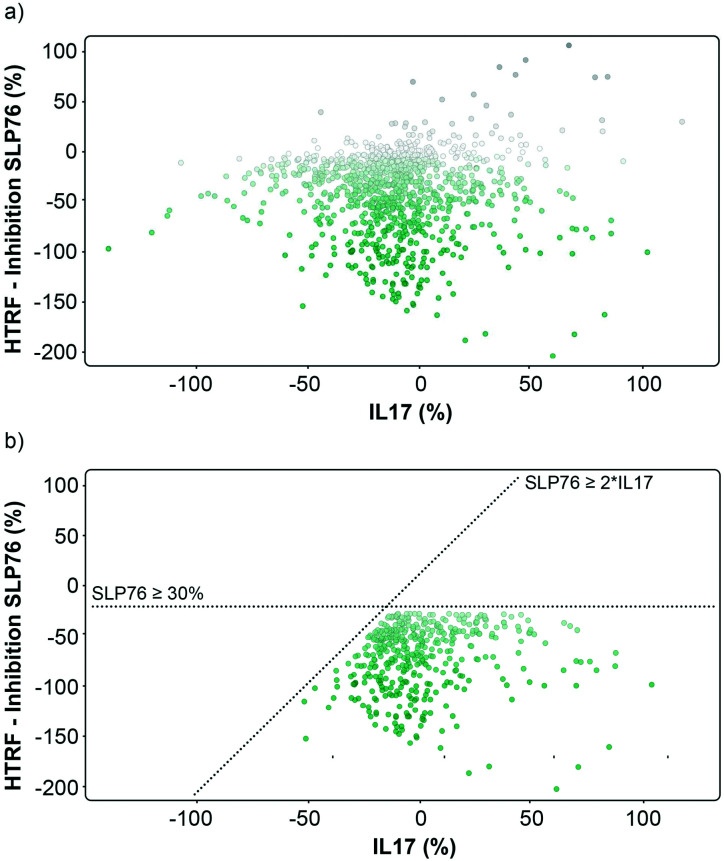
Graphical representation of the repeated 1136 hits tested on 14-3-3/SLP76 and PPI X. a) Representation of the hits expressed as percent inhibition against 14-3-3γ/SLP76 and IL17/IL17R. Potential 14-3-3/SLP76 stabilisers are represented as green dots. b) Selection conditions applied to the hits were to retain compounds that showed an effect on SLP76 ≥ −30% and simultaneously at least a two-fold greater stabilisation than IL17 PPI, SPL76 ≥ 2*IL17.

The cut-off expressed in number of standard deviations of the DMSO control from the mean was 4*SD_DMSO_ (or 30*SD_DMSO_ calculated using raw data). The cut-off chosen from the primary screen left us with 1136 hits, roughly 5% of the initial 20 000 molecules. Of these 1136 small molecules, 660 repeated in a confirmatory screen with the same cut-off threshold; roughly 3% of the initial 20 000 compounds. These 660 hits were subsequently tested in an assay using IL17/receptor as an unrelated PPI which shared the same FRET acceptor/donor pair as the 14-3-3γ/SLP76 system (Fig. 3a).

The matched FRET pair counter screen was specifically introduced to reduce the number of false positive hits and to focus on molecules that selectively stabilised the 14-3-3γ/SLP76 system. Using this counter screen, we were able to further refine compounds of most interest to 428 hits as summarised in Fig. 3b.

**Fig. 4:**
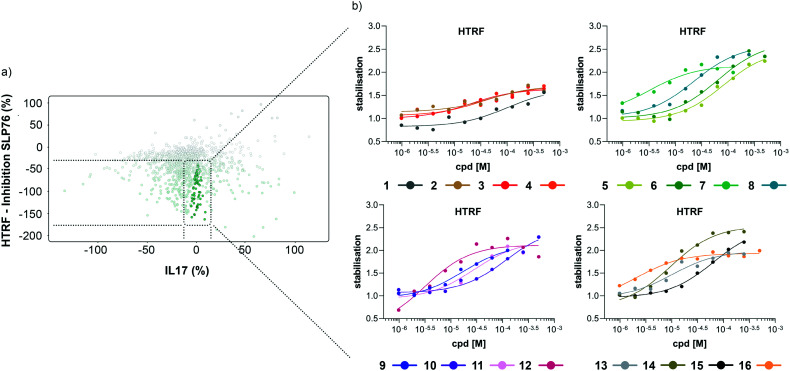
Representation of the 64 small molecules selected on the basis of SLP76 stabilisation and IL17/IL17R counter-screen and 14-3-3γ/SLP76 dose response curves for the best 16 compounds. a) Selection of 64 small molecules represented as green dots within dotted lines. b) Dose response curves of the best 16 out of the 64 compounds tested. Estimated EC50 values for every compound are reported in (Table 1).

The hits selected in this way were then ranked according to their inhibition ratio (% inhibition SLP76/% inhibition IL17), with the most selective compounds (highest ratios) prioritised for further study. This allowed us to further focus our efforts on a shortlist of 64 compounds which demonstrated little to no stabilisation of the counter-screen system with the results of this triage process summarised in Fig. 4a.

### HTRF dose–response assay on the selected 64 compounds

**Fig. 5:**
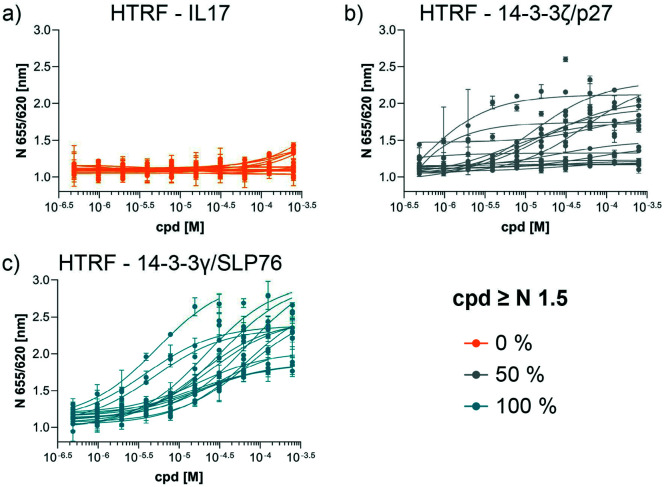
Comparison of the dose response assay of the 16 compounds on the systems IL17/IL17R, 14-3-3ζ/p27 and 14-3-3γ/SLP76. a) Dose response of the 16 compounds on IL17/IL17R. None of the compounds showed an effect on IL17/IL17R. b) Dose response of the 16 compounds on 14-3-3ζ/p27. 8 out 16 compounds showed an effect greater than 1.5 normalised response. c) All 16 compounds repeated on 14-3-3γ/SLP76.

The compounds which appeared selective in the single point screening were tested as dose responses on 14-3-3γ/SLP76 (Fig. 4b). Of the 64 tested in dose response, 30 did not show any dose–response effect. Of the remaining 34 compounds, 16 were selected as the most promising hits to follow up based on EC50 values (Table 1). All 16 compounds demonstrated a concentration dependent increase in HTRF signal as a result of the binding of the molecules to the 14-3-3/SLP76 complex. EC50 values estimated from the fitting curves were between 2.2 and 110 μM (Fig. 4b). These 16 compounds were retested in dose response using 10 mM DSMO stock solutions freshly prepared from solid and pleasingly they were all confirmed as stabilisers of the 14-3-3γ/SLP76 PPI system (Fig. 5c). To test whether the concentration-dependent effect would influence other PPI systems as well, the compounds were also tested on IL17/IL17R as an unrelated PPI (Fig. 5a) and on the 14-3-3ζ/p27 system (Fig. 5b) as an example of another 14-3-3 PPI. In all cases the same FRET pair was used to label the different protein systems.

**Table 1:** Summary table of the 16 compounds identified from the HTRF dose–response assay

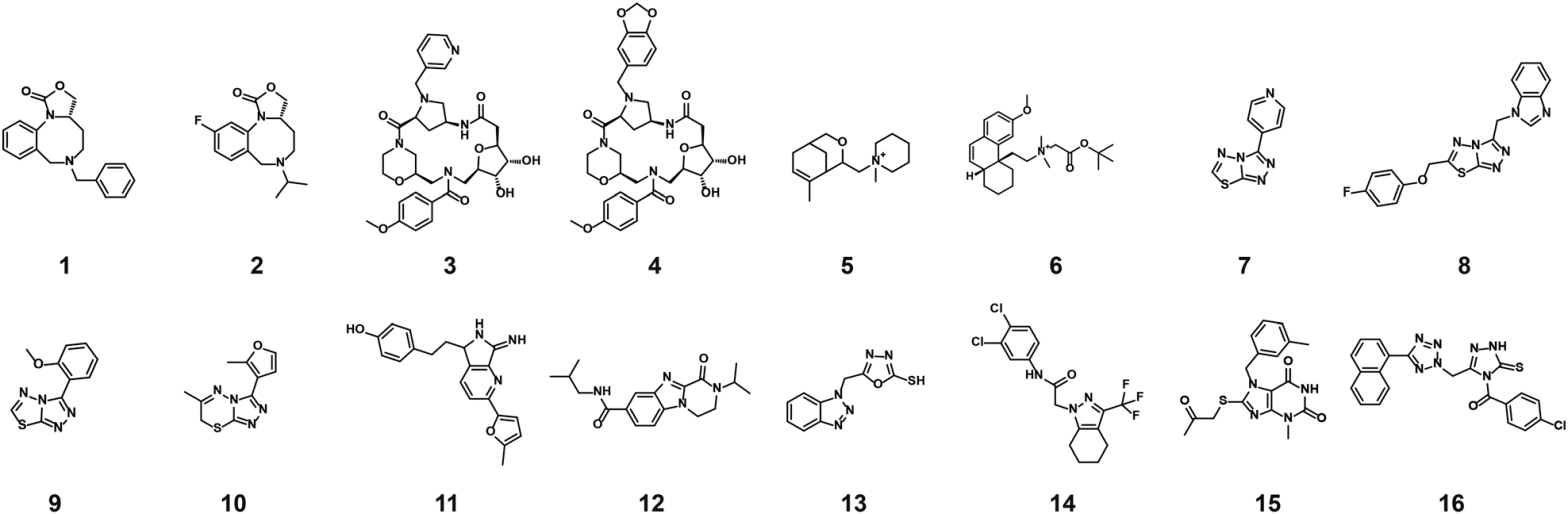				
Molecule	HTRF EC50 (μM)		HTRF *K*_D_ improvement	SPR RU (%) mass increase
	14-3-3γ/SLP76	14-3-3ζ/p27	14-3-3γ/SLP76	14-3-3γ/SLP76
**1**	19.1 ± 7.7	IA^a^	3.3×^c^	66.4
**2**	18.6 ± 5.5	IA^a^	4.1×^c^	67.6
**3**	14.4 ± 1.9	IA^a^	10×^c^	66.9
**4**	17.4 ± 4.9	IA^a^	3.4×^c^	57.1
**5**	71.1 ± 23.9	IA^a^	4.7×^c^	Ns^d^
**6**	33.5 ± 10.7	IA^a^	Nm^b^	Ns^d^
**7**	5.3 ± 1.9	IA^a^	4.4×^c^	57.7
**8**	69.1 ± 22.6	53.9 ± 17.1	3.5×^c^	61.2
**9**	24.5 ± 5.8	5.8 ± 2.2	11×^c^	58.7
**10**	70.1 ± 8.7	16.5 ± 10.6	1.4×^c^	Ns^d^
**11**	29.7 ± 21.8	128.9 ± 70.5	Nm^b^	Nm^b^
**12**	3.8 ± 1.2	0.43 ± 0.37	5.2×^c^	67.4
**13**	4.9 ± 1.1	IA^a^	1.1×^c^	Ns^d^
**14**	21.2 ± 11.3	12.6 ± 14.4	14.6×^c^	Ns^d^
**15**	17.1 ± 5.2	11.6 ± 6.1	Nm^b^	Nm^b^
**16**	1.2 ± 0.7	80.6 ± 53.4	1.8×^c^	Nm^b^

a IA: inactive no stabilisation observed in dose–response.

b Nm: not measured.

c *n*-Fold 14-3-3γ/SLP76 *K*_D_ improved in presence of increasing concentration of small molecule.

d Ns: no stabilisation observed in SPR.

None of the 16 compounds showed an effect on IL17/IL17R, as expected from the primary counter screen assay. However, eight compounds showed a dose–response effect on the 14-3-3ζ/p27 system that exceeded the normalised response of 1.5, with 2.0 being the theoretical maximal response. This evidence indicates that these compounds are not specifically selective for the 14-3-3γ/SLP76 PPI and provides an opportunity for further exploration. Although the key objective of this study was to find selective stabilisers of the 14-3-3γ/SLP76 interaction, the prospect of finding generic 14-3-3 PPI stabilisers would be of general benefit to research in this area. Furthermore, if the binding site of these compounds could be characterised it would open the possibility to rationally build in selectivity for different 14-3-3 protein–protein interactions through structure-based drug design.

### Orthogonal SPR confirmation on the selected 16 compounds

Of the 16 hit compounds 13 were tested in an orthogonal SPR assay in which each compound was flowed with SLP76 over 14-3-3γ immobilised on the chip. Runs at different concentrations of compound were compared to the runs in absence of compound. Of the 13 compounds tested this way, 8 showed a difference in off-rate indicative of increased binding of SLP76 to 14-3-3γ and were subsequently tested at multiple concentrations to establish a concentration-dependent effect (Fig. S2–S17†). All 8 compounds proved to be dose responsive leading to a concentration-dependent increase in mass on the chip. The effect of the compounds on 14-3-3γ/SLP76 was measured by calculating the percentage of SLP76 remained on the chip after the dissociation phase and immediately before: RU (%) = RU 5 s before dissociation/RU +240 s after dissociation. Dose–response curves were extrapolated from those sensorgrams plotting the RU (%) mass increase over the increase in compound concentrations.

### Analysis of hit compounds

The structures of all 16 hits and data from all assays used to triage the compounds are summarised in Table 1. Of the 16 compounds tested by HTRF in dose response, compounds **1**, **2**, **3**, **4**, **7**, **8**, **9**, **12** were orthogonally confirmed by SPR and of those, compounds **1**, **2**, **3**, **4**, **8** were selective for 14-3-3γ/SLP76 over 14-3-3ζ/p27 (Fig. S2–S5, S8–S10 and S13†). Ten of the sixteen hit compounds could be grouped into four structurally distinct chemotypes with the remaining 6 compounds singleton hits.

**Fig. 6:**
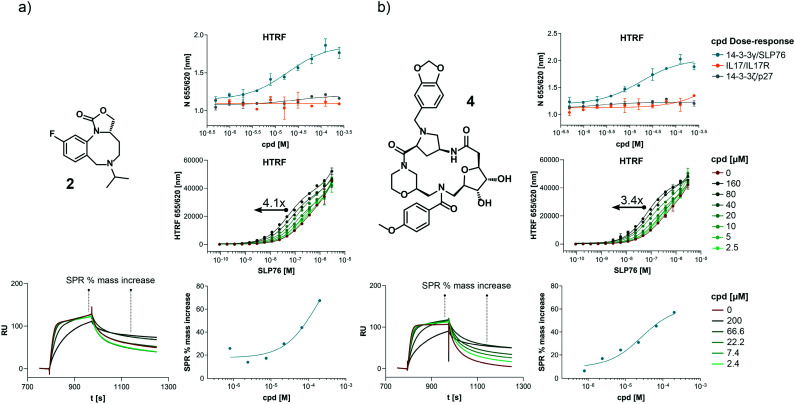
Combined set of assays example for the compound **2** and **4**. a) HTRF dose response comparison of the three systems used. HTRF dose ratio assay on the 14-3-3γ/SLP76-SH2 system. The arrow represents the increment in potency upon increasing compound concentration. Sensorgrams generated by SLP76-SH2 flowing over 14-3-3γ in absence (as reference) and in the presence of different concentration of compounds. The plot of the reference points taken from the sensogram used to extrapolate the SPR apparent EC50 is reported to the right. The reference points before and after the dissociation phase are highlighted in the sensograms (bottom left). b) The same set of assays are repeated on compound **4**.

Compounds **1** and **2** belong to an interesting benzo-fused tricyclic oxazolidinone system. Both compounds were selective for the 14-3-3γ/SLP76 interaction with similar activities and no observed stabilisation in the 14-3-3ζ/p27 HTRF assay. The stabilising effect of these compounds was orthogonally confirmed and equipotent by SPR (Fig. 6a). These oxazolidinones have good lead-like properties with low MW, PSA and good *A* log *P* (SI) which makes them excellent candidates for hit expansion. The presence of a chiral centre offers the prospect to investigate if there is an enantiomeric preference in stabilisation.

An equally interesting chemotype are the 16-membered macrocyclic compounds **3** and **4** which were also shown to be selective for the 14-3-3γ/SLP76 system as well as being orthogonally confirmed in the SPR assay (Fig. 6b). Although the macrocycles show very similar levels of activity in the 14-3-3γ/SLP76 HTRF assay to compounds **1** and **2**, they both appear to perform better in the SPR stabilisation assay with compound **3** showing one of the strongest effects in *K*_D_ improvement (10 fold) in the SLP76 HTRF assay across all compounds tested. The macrocycles lie outside the range of conventional lead-like properties with MW of 609 and 652 respectively, highly polarity (*A* log *P* −1.3 and −0.5) and with PSA around 150 which could impact cell permeability. However, this should not preclude a more detailed investigation and indeed these compounds could give valuable insight as tools for future structural studies.

The third group of hits, compounds **5** and **6**, are characterised by having a quaternary positively charged amine group. Both showed activity in the 14-3-3γ/SLP76 HTRF assay although with modest EC50s and were selective over 14-3-3ζ/p27. However, neither of these two compounds were confirmed in SPR with no observable stabilisation effect (Fig. S6 and S7†).

The fourth group of compounds: **7**, **8**, **9** and **10** all share a triazolothiadiazole heteroaromatic scaffold or in the case of compound **10** the closely related triazolothiadiazine structure. This set of compounds is especially attractive in that they have low MW with good molecular properties in fragment space and have significant potential for further exploration. It is also notable that these compounds form the largest structurally related cluster of SLP76 stabilisers from the UCB Diversity Deck. Compound **7** is the stand-out hit as a selective 14-3-3γ/SLP76 stabiliser with EC50 of 5.3 μM, a fourfold increase in *K*_D_ and no measurable modulation of the 14-3-3ζ/p27 system. Compound **7** also has a strong concentration dependence in off-rate in SPR when compared to other compounds in this group (*e.g.* compound **10** demonstrates no observable stabilisation in the SPR assay). With a MW of only 203 compound **7** demonstrates remarkable modulation of the 14-3-3γ/SLP76 system and is a priority for further focussed hit expansion. What is even more interesting is that the closely related analogues in this group are also able to stabilise the 14-3-3ζ/p27 PPI to a lesser or greater extent. This opens up the intriguing possibility that these compounds could be generic modulators of 14-3-3 protein–protein interactions, with the potential to tune-in selectivity for a specific protein pair. This speculation would of course require a larger confirmatory study to expand both the chemical matter around these scaffolds and the numbers of 14-3-3 PPIs tested. For these compounds to be general stabilisers of the 14-3-3 PPIs it is reasonable to expect that they are interacting with a conserved pocket on the 14-3-3 protein. Compound **8** is the closest analogue of **7** and is a weak and equipotent stabiliser of both the 14-3-3γ/SLP76 and 14-3-3ζ/p27 systems. Compounds **9** and **10** in contrast show a modest level of selectivity in stabilising 14-3-3ζ/p27 over 14-3-3γ/SLP76 (Fig. S10 and S11†).

The last group of compounds are six singleton hits compounds **11** to **16** which, with the exception of compound **13** are able to stabilise both the 14-3-3γ/SLP76 and 14-3-3ζ/p27 systems (Fig. S12–S17†). The dihydropyrazino[1,2-a]benzimidazole, compound **12**, is the most potent stabiliser of 14-3-3ζ/p27 tested with an EC50 of 0.43 μM, almost 9-fold selective over 14-3-3γ/SLP76 (EC50 of 3.2 μM). With ability to modulate both systems and with excellent molecular properties, compound **12** should be followed-up in greater detail. The last compound we would highlight is compound **14** which is another attractive chemical starting point for further investigation. Although this compound provides modest stabilisation EC50s for both the 14-3-3γ/SLP76 and 14-3-3ζ/p27 systems (EC50 21.2 μM and 16.6 μM respectively), it shows the highest improvement in SLP76 HTRF *K*_D_ at 14-fold. However, this stabilisation was not confirmed in the SPR assay. The full set of assays per compound is reported in the Supplementary Information.

## Material and methods

### Protein expression and purification

14-3-3γ and SLP76 were expressed respectively with a (His)_6_ and (His)_6_-SUMO tag in Nico21(DE3) competent cells, in 2TY media. The purification was carried out by affinity chromatography on nickel columns (HisTrap HP, 5 mL). The tags were cleaved with TEV or SUMO protease. The proteins were then loaded again on nickel columns to remove any uncleaved protein. A final purification step was performed loading the proteins on a size-exclusion chromatography column (HiLoad 26/600 Superdex 75 pg). All purification steps were performed on an ÄKTA pure protein purification system (Cytiva). SLP76 was phosphorylated *in vitro* by incubating overnight at room temperature in its storage buffer supplemented by 0.75 mM ATP, 20 mM MgCl_2_ and HPK1 kinase (Thermo Fisher Scientific) with a kinase : protein ratio of 1 : 3000. Phosphorylation status was checked by LC-MS. The expression, purification and phosphorylation of the SLP76 construct (SLP76-SH2) has been described before.^63^

### Protein labelling

14-3-3γ was labelled with Tb cryptate: LanthaScreen™ Amine Reactive Tb Chelate, (PV3582, ThermoFisher). SLP76 was labelled with AF647: Alexa Fluor™ 647 carboxylic acid, succinimidyl ester, A37573 (ThermoFisher). Before labelling, the protein buffers were exchanged to sodium carbonate buffer at pH 9.0 to allow deprotonation of amine groups. The proteins were subsequently diluted down to 50 μM with sodium carbonate buffer, and a 7-fold and 5-fold excess of Tb cryptate and AF647 were added to 14-3-3γ and SLP76 respectively. The mixtures were left in the dark for 1 h at room temperature. After labelling, the buffers were ultimately exchanged to assay buffer (50 mM Tris pH 7.5, 150 mM NaCl, 5 mM MgCl_2_, 0.05% v/v Tween-20). All the buffer exchange steps were performed with Zeba™ Spin Desalting Columns, 7 K MWCO, 0.5 mL (ThermoFisher) and P-10 Desalting Columns (Cytiva) for higher volumes.

### Homogeneous time-resolved fluorescence assay development and high throughput screening

384-Well logistic plates were prefilled with 2 μL of compounds at 2 mM, 100% DMSO, except for columns 1, 2, 23 and 24. Columns 1, 2 were filled with 2 μL of DMSO while columns 23, 24 were filled with 2 μL of the 14-3-3 inhibitor R18 at 20 μM using a Multidrop Dispenser (ThermoScientific). Next, 40 μL of protein mixture containing Tb-labelled 14-3-3γ at 1 nM and AF647 labelled SLP76 at 600 nM in assay buffer (50 mM Tris pH 7.5, 150 mM NaCl, 5 mM MgCl_2_, 0.05% v/v Tween-20) were added using a Multidrop Dispenser (ThermoScientific). Hence, the final assay concentrations were 14-3-3γ-Tb at 1 nM, SLP76-AF647 at 600 nM, compounds at 100 μM, R18 at 1 μM, and DMSO at 5% v/v. To perform the assay, 30 μL volumes were then transferred using a Biomek FX^P^ Automated Workstation (Beckman Coulter) into Corning 384-well 3574 assay plates. The plates were incubated at room temperature with gentle shaking for 4 h. Finally, the data were collected on a PHERAstar FSX plate reader (BMG Labtech) with an *λ*_ex_ 337 nm, *λ*_em_ 665 nm and 620 nm HTRF filter setting. The data were exported in IDBS ActivityBase (IDBS) where they were normalised setting the R18 negative control as 100% inhibition and the DMSO control as 0%. Negative percentage values were therefore associated with a stabilisation of the 14-3-3γ/SLP76 interaction. The threshold of −20% inhibition (or 20% stabilisation) was chosen to select the hits to follow up. The selected hits were retested on 14-3-3γ/SLP76 and were also tested against an unrelated protein pair (IL-17/receptor) in a similar assay setup. Only the ones that had an effect on 14-3-3γ/SLp76 and not on the IL-17/IL17 receptor system were selected. All the assays were performed as described above.

### Homogeneous time-resolved fluorescence dose–response and dose-ratio

384 logistic plates were prefilled with 40 μL of compounds at 10 mM, 100% DMSO in column 3. Columns 1, 2 and 4 to 22 were filled with 20 μL of 100% DMSO with a Multidrop Dispenser (ThermoScientific). 2-Fold dilutions of the compounds were carried over from column 3 to 22, with a CyBio FeliX liquid handler (analytikjena). 2 μL of R18 peptide at 20 μM was added to columns 23 and 24 of the Corning 384-well 3574 final assay plates with a Multidrop Dispenser (ThermoScientific). 40 μL of protein mixture, containing Tb labelled 14-3-3γ at 1 nM and AF647 labelled SLP76 at 600 nM in assay buffer (50 mM Tris pH 7.5, 150 mM NaCl, 5 mM MgCl_2_, 0.05% v/v Tween-20) were added with a Multidrop Dispenser (ThermoScientific) into the Corning 384-well 3574 final assay plates. 2 μL of compounds along with the DMSO control were then transferred with a CyBio FeliX liquid handler (analytikjena) from the 384 logistic plates to the final Corning 384-well 3574 assay plates. This resulted in final assay concentrations of: Tb labelled 14-3-3γ at 1 nM, AF647 labelled SLP76 at 600 nM, compound concentrations between 500 and 50 μM following a 2-fold dilution, and R18 peptide at 1 μM in 5% DMSO. The dose–response on the first 64 compounds was performed in singlet from a 500 μM top concentration. The repetition on the best 16 compounds was performed in duplicates from a 250 μM top concentration. A dose–response assay on an unrelated 14-3-3ζ/p27 HTRF protein pair was performed as described above. Incubation, data acquisition and normalisation were performed as described above. For EC50 estimation, the data were fitted with a “log(agonist) *vs.* response (three parameters)” model on GraphPad Prism version 8.1.1 for Windows, GraphPad Software, La Jolla California USA, www.graphpad.com. In the case of duplicates, each data point is the average of a duplicate measurement, standard deviation is reported as error bars.

Dose-ratio assays were carried out on Corning 384-well 3575 plates, serially diluting (2-fold) SLP76-AF647 in the presence of 14-3-3γ-Tb 1 nM from a top concentration of 3 μM. Each protein titration was performed in the presence of a fixed concentration of compound. A set of 8 titration curves per compound (160, 80, 40, 20, 10, 5, 2.5, 0 μM) were performed. Proteins and R18 peptide were diluted from their stock concentration in assay buffer (50 mM Tris pH 7.5, 150 mM NaCl, 5 mM MgCl_2_, 0.05% v/v Tween-20). Incubation and data acquisition were performed as described above. One titration series of SLP76-AF647 on 14-3-3γ was performed in the constant presence of R18 10 μM that completely inhibits the interaction. For data analysis the background FRET signal generated by the titration in the presence of R18 10 μM was removed from all values. *K*_D_ values for each curve were estimated and comparison with the different concentration of compounds were made. The data were fitted with a “One site – specific” model on GraphPad Prism version 8.1.1 for Windows, GraphPad Software, La Jolla California USA, www.graphpad.com. Each data point is the average of a duplicate measurement, standard deviation is reported as error bars.

### Surface Plasmon resonance

SPR experiments were performed on a Biacore T200 (Cytiva). The Sensor Chip NTA was validated as previously reported by Soini *et al.*^63^ A DMSO tolerance at 2% and 5% was performed in the same way (Fig. S1†).

For compounds testing, the compounds were preincubated with SLP76, in final DMSO concentration of 5%. The compounds were tested at a single concentration of 100 μM and at multiple concentrations following a 3-fold dilution from a top concentration of 100 μM with SLP76 at the saturation concentration of 250 nM. A second set of experiments were performed at multiple concentrations of compound following a 3-fold dilution from a top concentration of 200 μM with SLP76 around the *K*_D_ concentration of 40 nM. The SLP76/compound mixtures were flowed over the immobilised 14-3-3γ at 30 μL min^−1^ for 180 s before allowing dissociation for 5 minutes. After every cycle, the chip was washed with EDTA and Ni^2+^ and 14-3-3γ reinjected for the next cycle. The compound effects were measured by calculating the percentage of SLP76 remained on the chip after the dissociation phase and right before: RU (%) = RU _5 s before dissociation_/RU _+240 s after dissociation_. RU response points relative to +240 s after dissociation were also plotted and an apparent EC50 was estimated. The data were fitted with a “One site – specific” model on GraphPad Prism version 8.1.1 for Windows, GraphPad Software, La Jolla California USA, www.graphpad.com.

## Conclusions

In this study we have developed and performed a high-throughput screen of a chemically diverse 20 000 small molecule library to discover molecular glues of the 14-3-3γ/SLP76 protein–protein interaction. The use of counter screens with a related 14-3-3/p27 system and an unrelated PPI with matched fluorophores allowed us to reduce false positives and identify specific modulators of both 14-3-3 protein–protein interactions. The outcome was that we were able to find 16 novel stabilisers of the 14-3-3γ/SLP76 complex across a range of different chemotypes with good lead-like properties to support future research. Of these 16 hits, six compounds (compounds **1–5** and compound **7**) achieved our principal objective of selective stabilisation of 14-3-3γ/SLP76 with orthogonal confirmation in SPR and are a priority for further exploration. Of equal interest were a group of compounds that modulated both the 14-3-3 systems tested and offer the intriguing possibility that these molecules may be of general utility in the field of 14-3-3 research. These preliminary results already hint that selectivity may be achievable in this group of compounds with compound **12** one of the most interesting compounds for further investigation which stabilised the 14-3-3/p27 system with an EC50 of 430 nM.

To the best of our knowledge this is the first report in the field of 14-3-3 PPIs to use a phosphorylated protein construct in an HTS HTRF format. In our opinion, such protein constructs have greater relevance than the short synthetic phosphopeptides that have been extensively used to represent the client protein in the 14-3-3 field to date. Although these phosphopeptides remain valuable tools to enable rapid early investigation of new 14-3-3 PPIs, they have inherent limitations as screening systems to find modulators of these interactions. By contrast working with full-length/truncated versions of phosphorylated 14-3-3 binding partners substantially extends the opportunity to discover druggable binding sites beyond the 14-3-3 amphipathic binding pocket. In this regard we believe that the results in this study support wider use as an approach to find molecular glues of 14-3-3 protein–protein interactions. Of course, the novel 14-3-3 stabilisers we report in this paper remain early hits and should still be treated cautiously. There is a need to continue to characterise these compounds to understand where they bind and how they stabilise the 14-3-3 ternary complex. Our priority will be to use X-ray crystallography and NMR to locate and rationalise the binding of the small molecules identified here. This will open the path for future optimisation of these molecules to tune and increase their stabilisation effect and ultimately demonstrate a therapeutically useful function.

## Author contributions

CO, JD and SL conceptualised and supervised the project. MR performed formal analysis and developed the HTRF methodology. MW developed the SPR methodology. LS conducted the investigation and visualisation process and wrote the original draft. CO, JD, SL, MR, MW and LS reviewed and edited the manuscript. CO is co-founder of Ambagon Therapeutics.

## Conflicts of interest

There is no conflict of interest to declare.

## Supplementary Material

MD-012-D1MD00172H-s001
