# Supplementary material for: Identification of molecular glues of the SLP76/14-3-3 protein–protein interaction
Source: RSC Med Chem. 2021 Aug 2;12(9):1555–64. doi: 10.1039/d1md00172h (PMC8459327; doi:10.1039/d1md00172h)
Supplement: MD-012-D1MD00172H-s001 [file MD-012-D1MD00172H-s001.pdf]

Electronic Supplementary Material (ESI) for RSC Medicinal Chemistry.
This journal is © The Royal Society of Chemistry 2021

Identification of molecular glues of the SLP76 / 14-3-3 protein-protein interaction

Lorenzo Soini[a,d], Martin Redhead[b], Marta Westwood[e], Seppe Leysen[c], Jeremy Davis[d], and Christian Ottmann[a, *]

[a]Laboratory of Chemical Biology, Department of Biomedical Engineering and Institute for Complex Molecular Systems, Eindhoven University of Technology, Eindhoven, The Netherlands;

[b]Exscientia ltd, Schrodinger Building, Oxford Science Park, Oxford, OX44GE

[c]Department of Structural Biology and Biophysics, UCB Celltech, Slough, UK;

[d]Department of Chemistry, UCB Celltech, Slough, UK

[e]Structural Biology, Discovery, Charles River, Chesterford Research Park, UK

Corresponding authors:

Christian Ottmann

c.ottmann@tue.nl

Jeremy Davis

Jeremy.Davis@ucb.com

Supportive Information

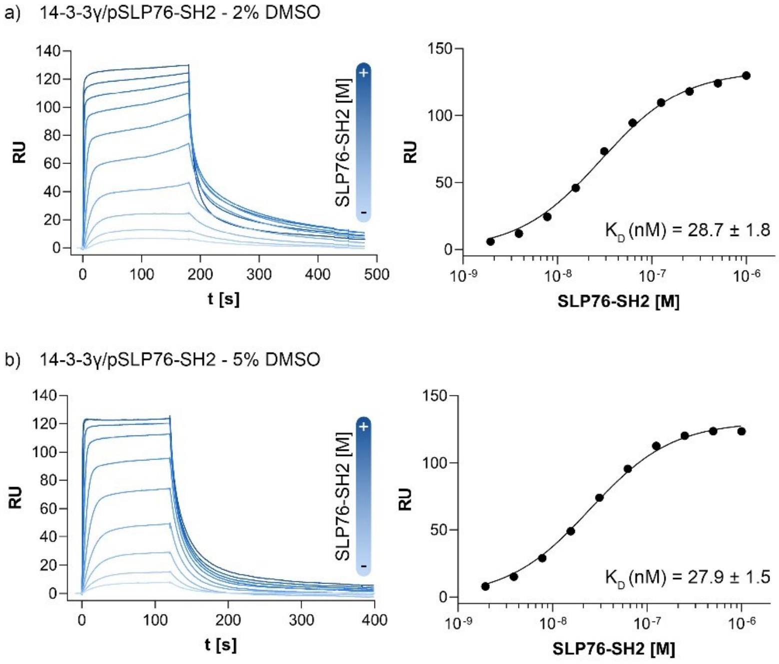

Figure S 1: SPR binding assay of SLP76-SH2 to 14-3-3γ at different DMSO concentrations. a) Sensorgram and respective binding curve extrapolated from reference points at equilibrium of SLP76-SH2 flowing over 14-3-3γ in the presence of 2% DMSO. b) Sensorgram and respective binding curve extrapolated from reference points at equilibrium of SLP76-SH2 flowing over 14-3-3γ in the presence of 5% DMSO. The K_D values has been extrapolated from the fitting model (One site - Specific binding model' on GraphPad Prism version 8.1.1 for Windows; GraphPad Software, www.graphpad.com.) and showed to be comparable the one to the other confirming a DMSO tolerance of the 14-3-3γ/SLP76-SH2 system in an SPR context. A K_D value calculated by Soini et al. in absence of DMSO (43 nM – ref.) is also comparable and along the previous results validate the chip surface for further studies.

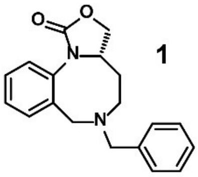

1

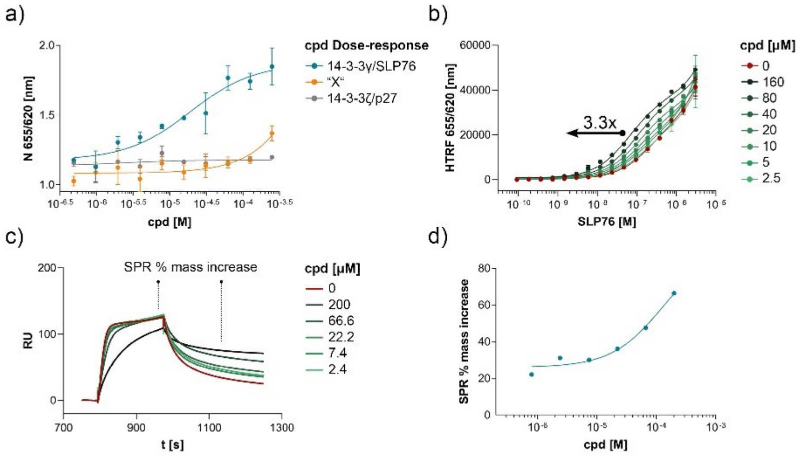

*Figure S 2: Set of assays for Compound **1**. a) HTRF dose-response comparison. b) HTRF dose-ratio assay with EC50 fold increase. c) Sensorgrams at different compound concentrations. d) Plot and fitting curve derived from the sensorgram reference points showed in c).*

2

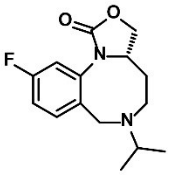

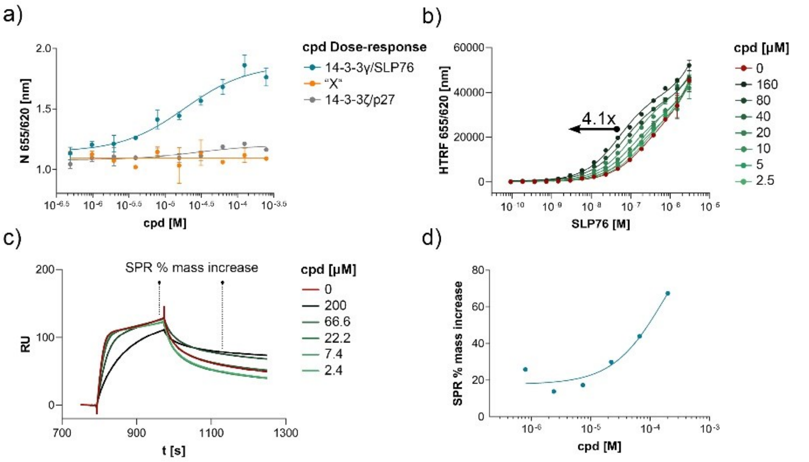

*Figure S 3: Set of assays for Compound **2**. a) HTRF dose-response comparison. b) HTRF dose-ratio assay with EC50 fold increase. c) Sensorgrams at different compound concentrations. d) Plot and fitting curve derived from the sensorgram reference points showed in c).*

3

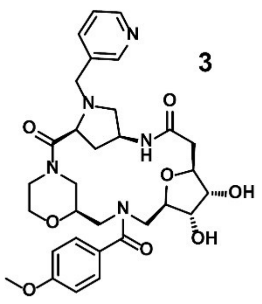

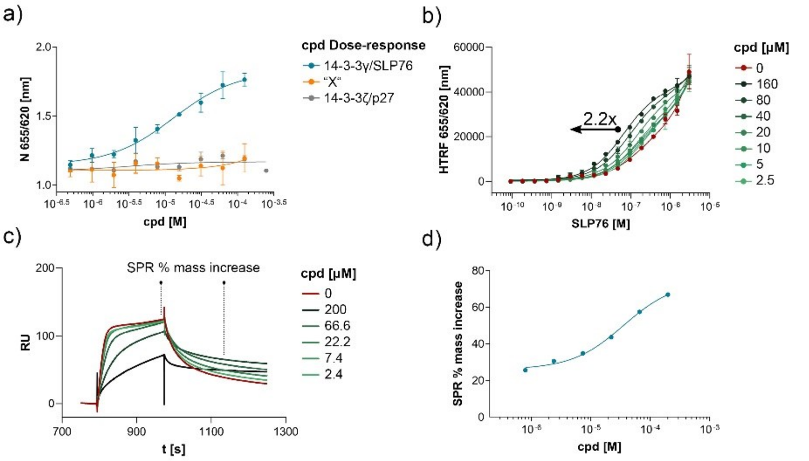

*Figure S 4: Set of assays for Compound **3**. a) HTRF dose-response comparison. b) HTRF dose-ratio assay with EC50 fold increase. c) Sensorgrams at different compound concentrations. d) Plot and fitting curve derived from the sensorgram reference points showed in c).*

4

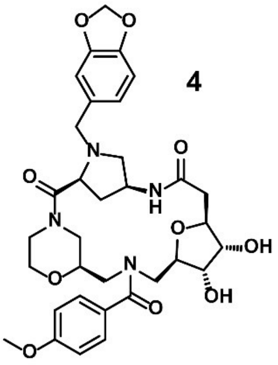

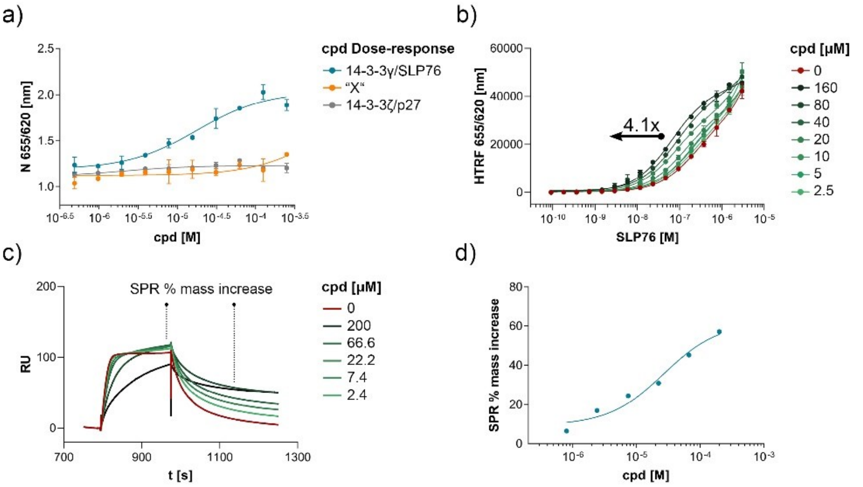

*Figure S 5: Set of assays for Compound **4**. a) HTRF dose-response comparison. b) HTRF dose-ratio assay with EC50 fold increase. c) Sensorgrams at different compound concentrations. d) Plot and fitting curve derived from the sensorgram reference points showed in c).*

5

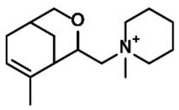

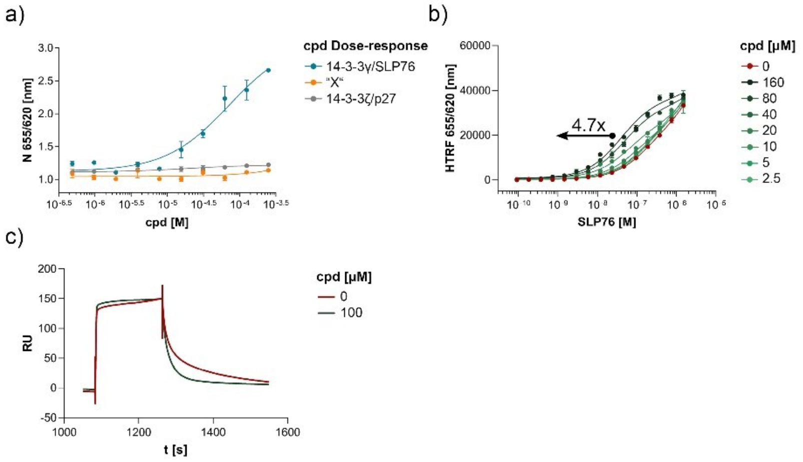

*Figure S 6: Set of assays for Compound **5**. a) HTRF dose-response comparison. b) HTRF dose-ratio assay with EC50 fold increase. c) Sensorgrams at different compound concentrations. d) Plot and fitting curve derived from the sensorgram reference points showed in c).*

6

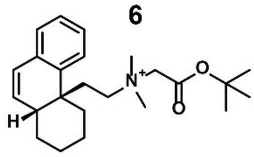

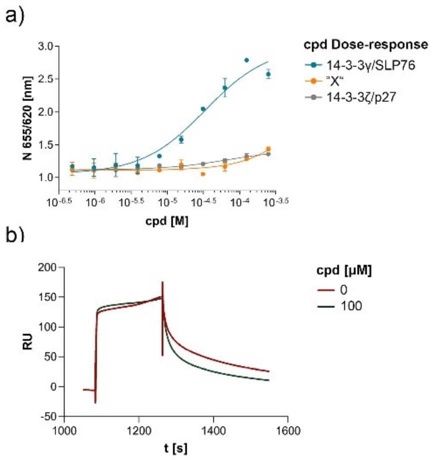

*Figure S 7: Set of assays for Compound **6**. a) HTRF dose-response comparison. b) HTRF dose-ratio assay with EC50 fold increase. c) Sensorgrams at different compound concentrations. d) Plot and fitting curve derived from the sensorgram reference points showed in c).*

7

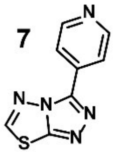

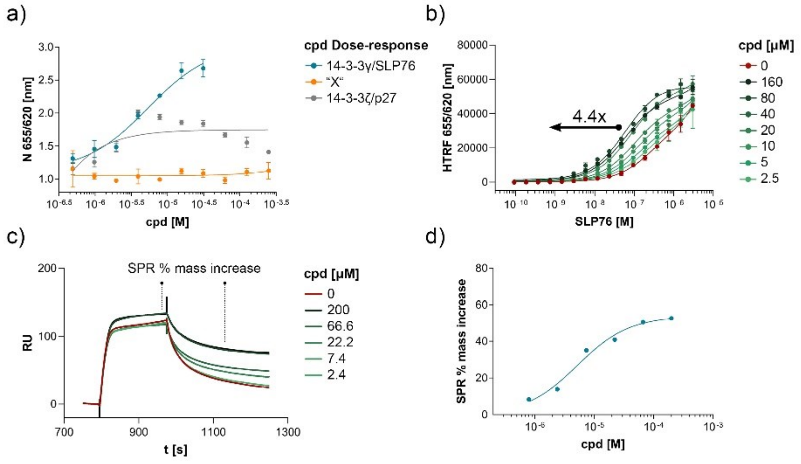

*Figure S 8: Set of assays for Compound **7**. a) HTRF dose-response comparison. b) HTRF dose-ratio assay with EC50 fold increase. c) Sensorgrams at different compound concentrations. d) Plot and fitting curve derived from the sensorgram reference points showed in c).*

8

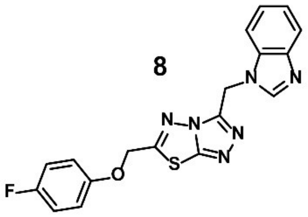

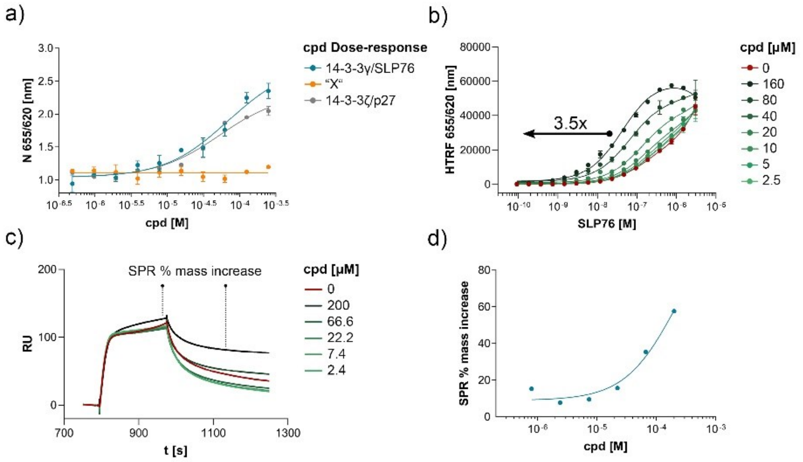

*Figure S 9: Set of assays for Compound **8**. a) HTRF dose-response comparison. b) HTRF dose-ratio assay with EC50 fold increase. c) Sensorgrams at different compound concentrations. d) Plot and fitting curve derived from the sensorgram reference points showed in c).*

9

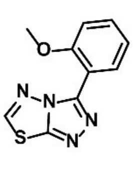

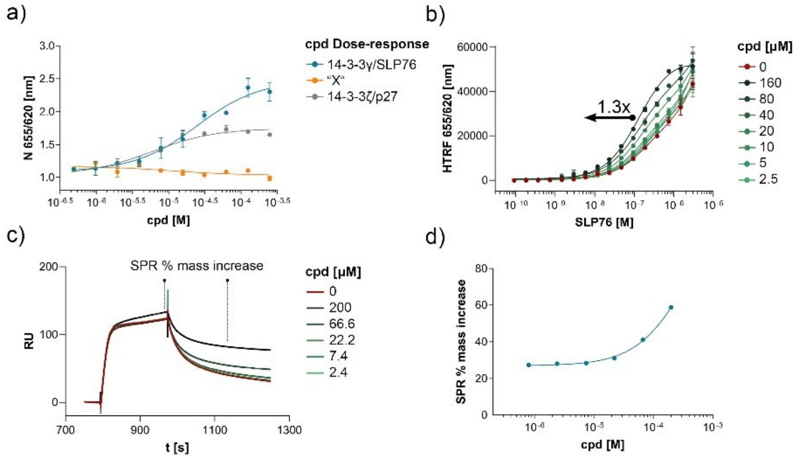

*Figure S 10: Set of assays for Compound **9**. a) HTRF dose-response comparison. b) HTRF dose-ratio assay with EC50 fold increase. c) Sensorgrams at different compound concentrations. d) Plot and fitting curve derived from the sensorgram reference points showed in c).*

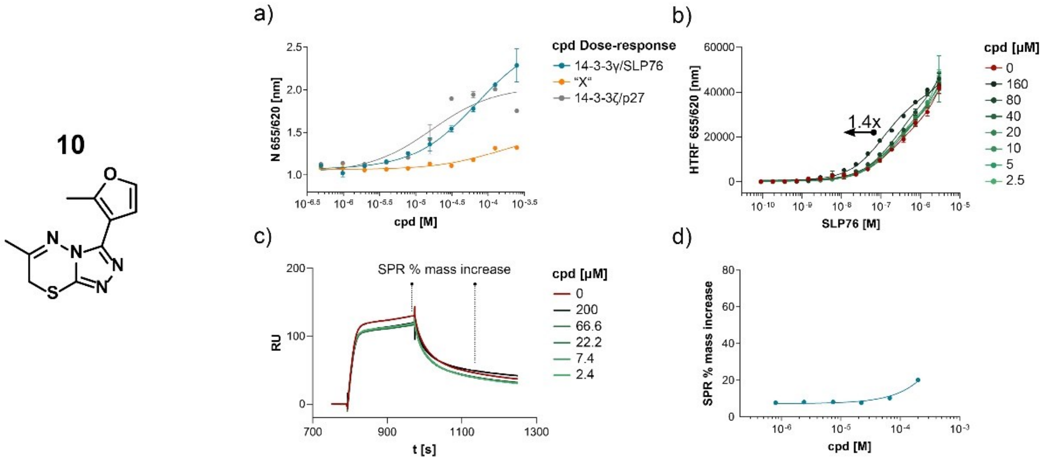

*Figure S 11: Set of assays for Compound **10**. a) HTRF dose-response comparison. b) HTRF dose-ratio assay with EC50 fold increase. c) Sensorgrams at different compound concentrations. d) Plot and fitting curve derived from the sensorgram reference points showed in c).*

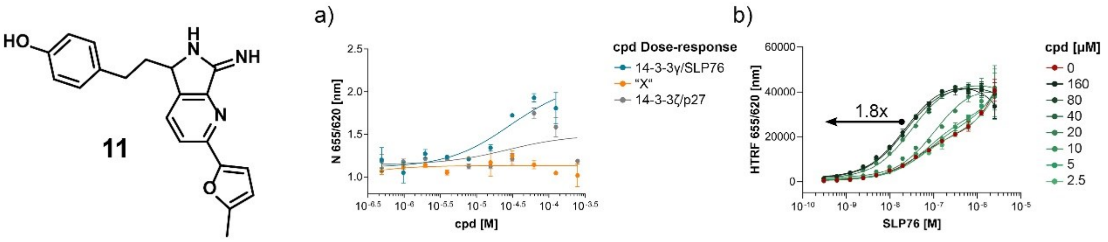

*Figure S 12: Set of assays for Compound **11**. a) HTRF dose-response comparison. b) HTRF dose-ratio assay with EC50 fold increase. c) Sensorgrams at different compound concentrations. d) Plot and fitting curve derived from the sensorgram reference points showed in c).*

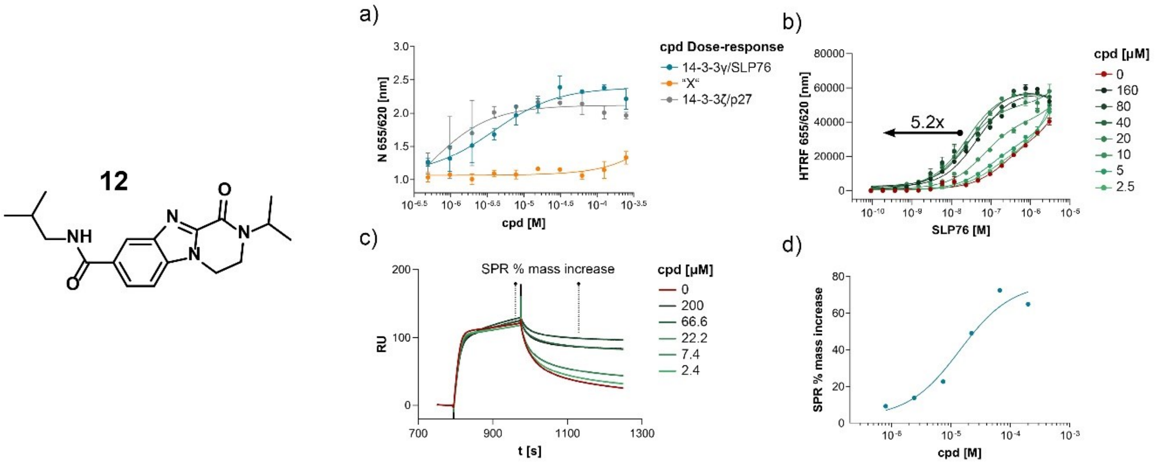

*Figure S 13: Set of assays for Compound **12**. a) HTRF dose-response comparison. b) HTRF dose-ratio assay with EC50 fold increase. c) Sensorgrams at different compound concentrations. d) Plot and fitting curve derived from the sensorgram reference points showed in c).*

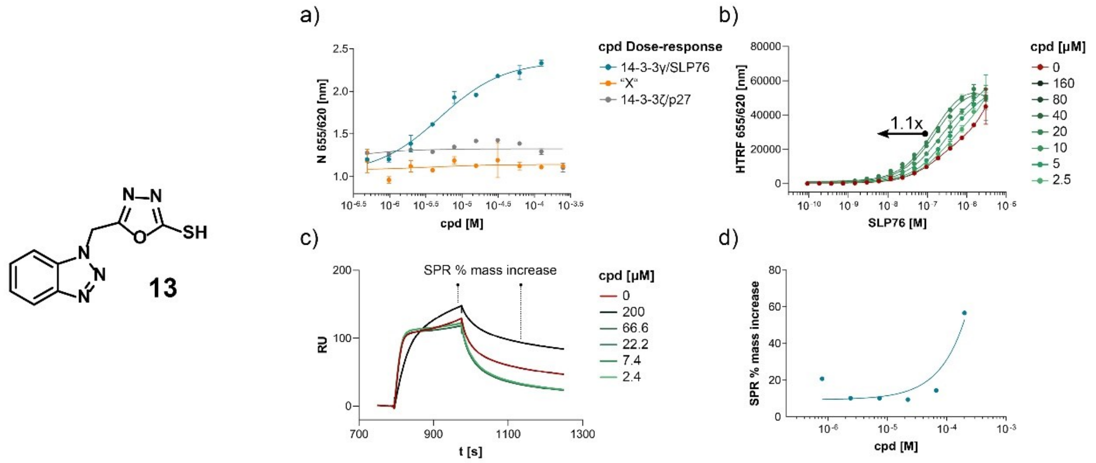

*Figure S 14: Set of assays for Compound **13**. a) HTRF dose-response comparison. b) HTRF dose-ratio assay with EC50 fold increase. c) Sensorgrams at different compound concentrations. d) Plot and fitting curve derived from the sensorgram reference points showed in c).*

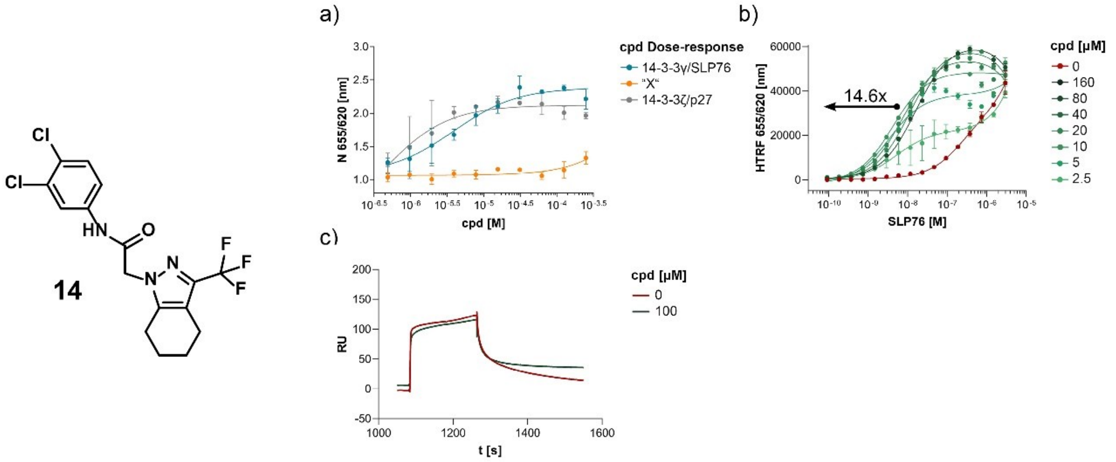

Figure S 15: Set of assays for Compound ***14****. a) HTRF dose-response comparison. b) HTRF dose-ratio assay with EC50 fold increase. c) Sensorgrams at different compound concentrations. d) Plot and fitting curve derived from the sensorgram reference points showed in c).*

15

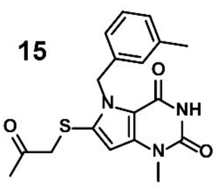

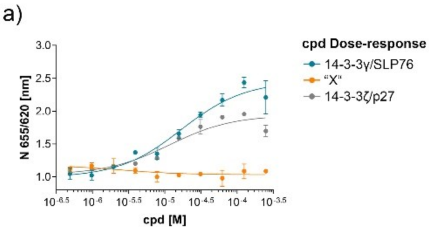

Figure S 16: Set of assays for Compound ***15****. a) HTRF dose-response comparison. b) HTRF dose-ratio assay with EC50 fold increase. c) Sensorgrams at different compound concentrations. d) Plot and fitting curve derived from the sensorgram reference points showed in c).*

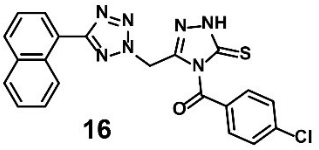

16

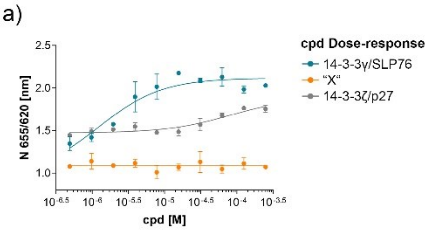

Figure S 17: Set of assays for Compound ***16****. a) HTRF dose-response comparison. b) HTRF dose-ratio assay with EC50 fold increase. c) Sensorgrams at different compound concentrations. d) Plot and fitting curve derived from the sensorgram reference points showed in c).*

Table S 1: Compounds molecular properties

Cpd. ID	MW (Da)	ALog P *	TPSA (Å²) ‡	CAS Number	IUPAC Name	Supplier
**1**	308.4	3.18	32.8	1797577-90-2	(6S)-9-benzyl-4-oxa-2,9-diazatricyclo[9.4.0.02,6]pentadeca-1(11),12,14-trien-3-one	AnalytiCon Discovery, GmbH
**2**	278.3	2.53	32.8	1796930-73-8	(6S)-14-fluoro-9-propan-2-yl-4-oxa-2,9-diazatricyclo[9.4.0.02,6]pentadeca-1(15),11,13-trien-3-one	AnalytiCon Discovery, GmbH
**3**	609.7	-1.32	154.2	n/a	(3S,6S,10S,11R,12S,13R,17R)-11,12-dihydroxy-15-(4-methoxybenzoyl)-4-(pyridin-3-ylmethyl)-18,22-dioxa-1,4,7,15-tetrazatetracyclo[15.3.1.13,6.110,13]tricosane-2,8-dione	AnalytiCon Discovery, GmbH
**4**	652.7	-0.41	159.5	n/a	(3S,6S,10S,11R,12S,13R,17R)-4-(1,3-benzodioxol-5-ylmethyl)-11,12-dihydroxy-15-(4-methoxybenzoyl)-18,22-dioxa-1,4,7,15-tetrazatetracyclo[15.3.1.13,6.110,13]tricosane-2,8-dione	AnalytiCon Discovery, GmbH
**5**	250.4	1.42	9.2	738575-09-2	1-methyl-1-[(8-methyl-3-oxabicyclo[3.3.1]non-7-en-2-yl)methyl]piperidin-1-ium	ChemBridge Corporation
**6**	400.5	3.32	35.5	692728-26-0	2-[(4aS,10aR)-6-methoxy-2,3,4,10a-tetrahydro-1H-phenanthren-4a-yl]ethyl-dimethyl-[2-[(2-methylpropan-2-yl)oxy]-2-oxoethyl]azanium	Chemieliva Pharmaceutical Product List
**7**	203.2	0.30	84.2	133847-06-0	3-pyridin-4-yl-[1,2,4]triazolo[3,4-b][1,3,4]thiadiazole	ChemBridge Corporation
**8**	380.4	2.99	98.3	791806-06-9	3-(benzimidazol-1-ylmethyl)-6-[(4-fluorophenoxy)methyl]-[1,2,4]triazolo[3,4-b][1,3,4]thiadiazole	Specs
**9**	232.3	1.44	80.5	1432902-04-9	3-(2-methoxyphenyl)-[1,2,4]triazolo[3,4-b][1,3,4]thiadiazole	Specs
**10**	234.3	1.29	81.5	892690-26-5	6-methyl-3-(2-methylfuran-3-yl)-7H-[1,2,4]triazolo[3,4-b][1,3,4]thiadiazine	Specs
**11**	333.4	3.76	82.1	n/a	4-[2-[7-imino-2-(5-methylfuran-2-yl)-5,6-dihydropyrrolo[3,4-b]pyridin-5-yl]ethyl]phenol	Chempartner
**12**	328.4	2.32	67.2	n/a	N-(2-methylpropyl)-1-oxo-2-propan-2-yl-3,4-dihydropyrazino[1,2-a]benzimidazole-8-carboxamide	ChemDiv
**13**	233.2	1.69	108.4	136715-68-9	5-(benzotriazol-1-ylmethyl)-1,3,4-oxadiazole-2-thiol	Maybridge, Ltd.
**14**	392.2	5.20	46.9	332943-31-4	N-(3,4-dichlorophenyl)-2-[3-(trifluoromethyl)-4,5,6,7-tetrahydroindazol-1-yl]acetamide	Specs
**15**	358.4	2.43	109.6	303973-50-4	3-methyl-7-[(3-methylphenyl)methyl]-8-(2-oxopropylsulfanyl)purine-2,6-dione	ENAMINE Ltd.
**16**	447.9	4.65	120.4	144338-89-6	(4-chlorophenyl)-[3-[(5-naphthalen-1-yltetrazol-2-yl)methyl]-5-sulfanylidene-1H-1,2,4-triazol-4-yl]methanone	Specs

* AlogP, calculated in Pipeline Pilot (Dassault Systemes) v 19; ‡ Topological Polar Surface Area, calculated in Piepline Pilot (Dassault Systemes) v 19
